# Effect of PPD inoculation on serum cytokine profiles in latent tuberculosis infection populations

**DOI:** 10.3389/fcimb.2025.1731603

**Published:** 2025-12-19

**Authors:** Wenjing Chang, Dingyong Sun, Yanqiu Zhang, Shaoping Ji, Shaohua Wang, Weidong Wang, Xiaoguang Ma, Danwei Zheng, Ruyue Su, Yankun Zhu, Jie Shi, Linqi Diao

**Affiliations:** 1 Henan Center for Disease Control and Prevention, Zhengzhou, China; 2 Center for Molecular Medicine, Zhengzhou Health College, Zhengzhou, China

**Keywords:** latent tuberculosis infection, PPD, cytokines, immune response, immunological monitoring

## Abstract

This study aims to investigate the impact of PPD skin test administration on serum cytokine levels in individuals with latent tuberculosis infection (LTBI) compared to healthy controls. Through quantitative analysis of multiple cytokines in serum samples before and after testing, significant differences were observed in cytokine level changes among LTBI individuals following PPD stimulation. The results indicate that prior to PPD administration, there were no significant differences in cytokine levels between the LTBI group and healthy controls. However, following PPD testing, individuals with LTBI exhibited significantly elevated levels of β-NGF, Eotaxin, G-CSF, GRO-α, IL-10, IL-17A, IL-1α, IL-1β, IL-2, IL-3, IL-4, IL-6, IP-10, LIF, and SDF-1α, while MCP-1 levels were significantly reduced compared to those in healthy controls. Comparative analysis of different population groups before and after PPD skin test administration revealed no significant changes in serum levels of SCGF-β, G-CSF, MCP-3, or IL-3 among healthy controls. In contrast, individuals with latent tuberculosis infection (LTBI) exhibited significantly elevated levels of G-CSF, MCP-3, and IL-3, while SCGF-β levels were markedly reduced. This study provides novel insights into the differential immune responses to PPD testing in latent tuberculosis infection and identifies potential biomarkers for early monitoring of immune activation in tuberculosis infection.

## 1 Introduction

Tuberculosis (TB) is a chronic infectious disease caused by *Mycobacterium tuberculosis*. Latent Tuberculosis Infection (LTBI) refers to the presence of the pathogen in the body without progression to active disease. According to the 2024 WHO report, more than one-quarter of the global population is infected with *Mycobacterium tuberculosis*, representing individuals with latent tuberculosis infection who do not exhibit clinical symptoms (Goletti et al., 2025). Although asymptomatic, approximately 5% of individuals with LTBI may progress to active TB within two years of infection, particularly under conditions such as immunosuppression (Keane et al., 2001; Wagner et al., 2008). However, the majority of infected individuals remain in a stable, asymptomatic state—likely due to effective host immune responses that control bacterial replication. Understanding the mechanisms underlying protective immunity against TB is essential for the development of novel diagnostic approaches, therapeutic strategies, and vaccine candidates (Sutherland et al., 2010). Therefore, timely diagnosis and monitoring of immune status in individuals with latent infection are of significant clinical importance.

In high tuberculosis burden countries, a cost-effective two-step diagnostic strategy for tuberculosis infection is widely implemented: initial screening using the tuberculin skin test (TST), followed by confirmatory testing with interferon-gamma release assays (IGRA) (Zyl-Smit et al., 2009). The TST, which employs purified protein derivative (PPD) extracted from *Mycobacterium tuberculosis* (Mtb), has long been the method of choice for diagnosing latent tuberculosis infection (LTBI), particularly in large-scale population screenings. PPD contains approximately 200 antigenic components—proteins derived from mycobacterial culture filtrates—and shares substantial antigenic homology with the Bacillus Calmette–Guérin (BCG) vaccine (Wagner et al., 2008). Following PPD administration, the host mounts an immune response mediated by the release of various cytokines. PPD induces a type IV delayed-type hypersensitivity (DTH) reaction through activation of the cellular immune system (Wagner et al., 2008), serving not only to detect prior Mtb infection but also to assess the immunogenicity and protective efficacy of vaccines such as BCG.

A growing body of evidence indicates that cytokines serve as pivotal signaling molecules in the immune system of individuals with latent tuberculosis infection (LTBI), regulating the activation, proliferation, and differentiation of immune cells, as well as modulating the magnitude and phenotype of immune responses. T cells are the primary mediators of adaptive immunity. In mammals, T cells express coreceptors: CD4 is expressed by T helper (Th) cell lineages, whereas CD8 is expressed by cytotoxic T cell lineages (De Obaldia and Bhandoola, 2015; Mu et al., 2022). Upon activation by antigen-presenting cells (APCs), CD4^+^ T cells can differentiate into distinct subsets, including T helper 1 (Th1), Th2, Th17, Th22, and regulatory T cells (Treg) (Ye et al., 2012; Tang et al., 2023), each producing a characteristic profile of cytokines associated with specific immune functions. Previous studies have demonstrated that cytokines such as IFN-γ and TNF-α play essential roles in host defense against *Mycobacterium tuberculosis* infection. The tuberculin skin test (TST) is widely recognized to induce a localized delayed-type hypersensitivity reaction; induration and erythema at the injection site constitute a positive result, indicating the presence of antigen-specific, sensitized Th1 cells. However, the effects of purified protein derivative (PPD) administration on systemic cytokine profiles in peripheral blood and its potential modulation of other Th cell-mediated immune responses in LTBI remain insufficiently characterized.

In this study, we aim to investigate the impact of PPD administration on cytokine profiles in the serum of individuals with latent tuberculosis infection (LTBI), as well as to elucidate the regulatory mechanisms underlying immune responses mediated by distinct T-cell subsets. Furthermore, we seek to explore the potential immunomodulatory effects of the tuberculin skin test (TST) in the context of *Mycobacterium tuberculosis* infection. These findings are expected to enhance understanding of the immunopathological features of LTBI and may provide valuable insights for the early diagnosis and management of tuberculosis.

## 2 Materials and methods

### 2.1 Study population

This study enrolled a total of 37 participants, who were divided into two groups: 21 individuals with latent tuberculosis infection (LTBI) and 16 healthy controls (normal group).

#### 2.1.1 Inclusion criteria

① Age ≥ 15 years; ② Provision of written informed consent and willingness to comply with all study procedures; ③ Positive tuberculin skin test (TST), defined as an induration diameter of at least 10 mm, and absence of active tuberculosis for inclusion in the LTBI group.

#### 2.1.2 Exclusion criteria

① Diagnosis of active pulmonary or extrapulmonary tuberculosis; ② Pregnancy, lactation, or planned pregnancy during the study period among female participants; ③ HIV infection; ④ Presence of diabetes mellitus; ⑤ Existing joint inflammation or other severe systemic comorbidities; ⑥ Use of glucocorticoids or immunosuppressive agents within the past 3 months; ⑦ History of psychiatric disorders that may compromise compliance or data interpretation; ⑧ Any condition considered unsuitable for participation by the principal investigator.

### 2.2 Sample collection and preservation

Blood samples were collected from participants in both groups at two time points: before PPD skin test administration (0 h) and after PPD skin test administration (48–72 h). Venous whole blood was collected in the morning under fasting conditions into 5 mL standard vacuum blood collection tubes. The tubes were then stored at 4 °C to allow complete clot formation. Following clotting, samples were centrifuged at 1712 × g for 10 minutes, and the resulting serum was carefully aliquoted into 1.5 mL microcentrifuge tubes (EP tubes) and immediately stored at –80 °C to ensure long-term stability.

### 2.3 Cytokine detection

All serum samples were retrieved from the storage facility and analyzed using the Luminex assay to quantify 48 cytokines: β-NGF, CTACK, Eotaxin, FGF basic, G-CSF, GM-CSF, GRO-α, HGF, IFN-α2, IFNγ, IL-1α, IL-1β, IL-1ra, IL-2, IL-2Ra, IL-3, IL-4, IL-5, IL-6, IL-7, IL-8, IL-9, IL-10, IL-12(p70), IL-12(p40), IL-13, IL-15, IL-16, IL-17A, IL-18, IP-10, LIF, M-CSF, MCP-1, MCP-3, MIF, MIG, MIP-1α, MIP-1β, PDGF-ββ, RANTES, SCF, SCGF-β, SDF-1α, TNF-α, TNF-β, TRAIL, and VEGF. The analyses were completed using the 96-well Bio-Plex Pro™ Human Cytokine 48-Plex Panel (Bio-Rad, Hercules, California, USA). According to the manufacturer’s instructions, cytokine concentrations were quantified using the Bio-Plex 200 System (Bio-Rad) with serial dilution methods. Detailed operational procedures and kit specifications are available in the official product manual at the following URL: https://www.bio-rad.com/sites/default/files/webroot/web/pdf/lsr/literature/10000092045.pdf. Samples with cytokine levels below the detection limit were assigned a value equal to zero, while those exceeding the upper detection limit were assigned the maximum standard concentration.

### 2.4 Statistical analysis

Of the 48 cytokines analyzed, GM-CSF, IL-5, IL-15, and VEGF were excluded from analysis because their concentrations were below the minimum detectable level or undetectable in more than 50% of samples. The remaining 44 cytokines were analyzed using SPSS 27.0 statistical software. The normality of data distribution was assessed using the Shapiro-Wilk test. Independent samples with a normal distribution were analyzed using the independent-samples t-test, while non-normally distributed data were evaluated using the Mann-Whitney U test (a non-parametric method). For paired samples, those with a normal distribution were analyzed using the paired-samples t-test, whereas non-normally distributed paired data were assessed using the Wilcoxon signed-rank test. A p-value < 0.05 was considered statistically significant. The false discovery rate (FDR) refers to the expected proportion of false positives among all statistically significant results. In this study, FDR correction was performed using both the Benjamini-Hochberg (BH) procedure and the p.adjust function in R 4.3.2. Cytokines meeting both criteria—p < 0.05 and FDR < 0.05—were considered significantly differentially expressed. The resulting differentially expressed cytokine profiles were visualized using GraphPad Prism 10.1.2.

## 3 Results

### 3.1 Baseline characteristics of the normal control and LTBI groups

**Table 1** summarizes the baseline characteristics of the two study populations. The chi-square test was used to compare gender distribution, while the independent samples non-parametric test (Mann-Whitney U test) was applied for the analysis of age and Body Mass Index (BMI). Fisher’s exact test was employed to assess comorbidities, history of BCG vaccination, and presence of a BCG scar. The results indicate no statistically significant differences between the groups with respect to ethnicity, gender, age, BMI, comorbidities, BCG vaccination history, or BCG scar presence (all p > 0.05). Detailed data are presented in **Table 1**.

**Table 1:** Baseline characteristics of the two study groups.

Variable	Group Category	Normal group	LTBI group	Test statistic (t/U)	p value
Ethnicity	Han	16	21	–	–
	Other	0	0		
Gender	Male	5	11	1.652	0.199
	Female	11	10		
Age		53.50(47.50, 56.00)	55.00(49.50, 62.50)	-0.783	0.434
Body Mass Index(BMI)		25.306(22.04, 28.831)	24.802(21.87, 27.871)	-0.399	0.690
Comorbidities				*	0.999
	None	14	16		
	Hypertension	1	2		
	Cardiovascular	1	2		
	Premature beats	0	1		
History of BCG Vaccination				*	<0.001
	Yes	13	19	*	0.634
	No	0	0		
	Unknown	3	2		
BCG vaccination scar				*	0.999
	Scar Present	12	15		
	Scar Absent	4	6		

*: Fisher’s Exact Test was used for categorical variables; no test statistic is reported due to complete separation or small expected frequencies.

### 3.2 Comparison of serum cytokine levels between healthy controls and LTBI subjects prior to PPD administration

Prior to PPD skin test administration, serum cytokine levels were compared between the healthy control group and the latent tuberculosis infection (LTBI) group. Among the analyzed cytokines, CTACK, G-CSF, HGF, IL-2Ra, IL-18, MIP-1β, RANTES, SCGF-β, TNF-α, TNF-β, and TRAIL exhibited a normal distribution (P > 0.05) and were analyzed using independent-samples t-tests. The remaining 33 cytokines deviated from normality (P < 0.05) and were therefore evaluated using the Wilcoxon rank-sum test, as detailed in **Table 2**.

**Table 2:** Comparison of cytokine levels between the control and LTBI groups prior to PPD administration.

Cytokine	Normal group mean ± SD/Median [P25-P75]	LTBI group mean ± SD/Median [P25-P75]	Test statistic (t/U)	p value	q value	Effect size (Cohen’s d/r)	95% CI for effect size
β-NGF	0.2(0,2.76)	1.46(0.14,2.86)	131	0.249	0.606	0.187	(0.01, 0.49)
CTACK	684.22 ± 268.14	1019.64 ± 477.40	-2.708	0.011	0.132	-0.836	(-1.509, -0.151)
Eotaxin	35.72(23.19,120.08)	91.68(66.4,172.79)	77	0.005	0.11	0.456	(0.119, 0.714)
FGF basic	18.66(0.38,37.97)	32.65(1.53,36.42)	161.5	0.841	0.906	0.03	(0.003, 0.407)
G-CSF	52.11 ± 24.77	50.81 ± 20.10	0.176	0.861	0.906	0.058	(-0.593, 0.709)
GRO-a	827.36(132.46,1219.17)	1134.25(861.59,1274.84)	105	0.053	0.365	0.315	(0.053, 0.599)
HGF	426.42 ± 133.51	451.69 ± 100.24	-0.658	0.515	0.708	-0.218	(-0.869, 0.435)
IFN-a2	0(0,6.17)	4.74(0,6.37)	142.5	0.404	0.652	0.134	(0.003, 0.444)
IFN-γ	39.3(26.87,50.58)	39.39(35.15,46.26)	155.5	0.701	0.827	0.06	(0.003, 0.411)
IL-10	2.21(0.64,12.76)	11.45(0.76,13.42)	149.5	0.569	0.736	0.091	(0.005, 0.402)
IL-12(p40)	39.06(33.63,53.16)	36.33(30.99,53.16)	154	0.666	0.827	0.068	(0.003, 0.395)
IL-12(p70)	3.85(0.04,7.42)	5.68(0.53,6.43)	161	0.829	0.906	0.033	(0.003, 0.41)
IL-13	1.92(0.27,2.86)	2.3(1.56,3.41)	120	0.14	0.583	0.24	(0.01, 0.53)
IL-16	42.89(35.4,56.62)	38.96(27.53,56.63)	135	0.312	0.606	0.164	(0.008, 0.479)
IL-17A	6.02(3.23,19.02)	18.28(4.93,20.25)	122	0.157	0.583	0.23	(0.015, 0.516)
IL-18	49.24 ± 24.99	64.10 ± 21.09	-1.961	0.058	0.365	-0.651	(-1.314, 0.022)
IL-1a	19.67(6,40.62)	35.86(11.51,42.98)	131.5	0.262	0.606	0.182	(0.008, 0.489)
IL-1β	2.38(0.77,6.31)	5.39(1.88,7.09)	121.5	0.153	0.583	0.232	(0.013, 0.526)
IL-1ra	207.79(182.1,248.11)	182.1(142.66,240.24)	134	0.296	0.606	0.169	(0.005, 0.468)
IL-2	0(0,7.32)	5.98(0,7.32)	146.5	0.487	0.708	0.112	(0.003, 0.427)
IL-2Ra	55.02 ± 20.38	57.77 ± 21.01	-0.401	0.691	0.827	-0.133	(-0.783, 0.519)
IL-3	0.38(0.03,0.68)	0.35(0.12,0.68)	162.5	0.865	0.906	0.025	(0.003, 0.352)
IL-4	0.66(0,1.95)	1.67(0.68,2.02)	125	0.186	0.606	0.215	(0.01, 0.512)
IL-6	0(0,2.91)	1.79(0,2.97)	137.5	0.32	0.606	0.161	(0.008, 0.486)
IL-7	18.76(1.32,28.06)	21.65(12.32,31.03)	141.5	0.415	0.652	0.132	(0.005, 0.459)
IL-8	8.87(5.61,11.72)	8.85(6.97,10.39)	165.5	0.939	0.939	0.01	(0.005, 0.372)
IL-9	270.41(113.31,561.07)	394.03(242.66,526.35)	132	0.27	0.606	0.179	(0.013, 0.507)
IP-10	268.78(195.05,478.46)	507.01(327.19,830.82)	77	0.005	0.11	0.456	(0.174, 0.699)
LIF	20.34(9.68,70.11)	53.6(13.81,72.29)	137	0.34	0.606	0.154	(0.005, 0.498)
MCP-1(MCAF)	46.2(31.2,72)	24.21(18.14,39.41)	86	0.012	0.132	0.411	(0.129, 0.679)
MCP-3	0.96(0,3.2)	3.02(0,3.49)	156.5	0.714	0.827	0.058	(0.003, 0.412)
M-CSF	15.32(10.97,17.67)	14.6(9.73,18.19)	164.5	0.915	0.936	0.015	(0.003, 0.366)
MIF	340.03(263.91,522.52)	425.95(275.89,600.63)	149	0.56	0.736	0.093	(0.008, 0.437)
MIG	238.06(187.57,314.65)	286.13(172.26,453.59)	138	0.358	0.606	0.149	(0.003, 0.452)
MIP-1a	2.16(1.71,2.81)	1.79(1.39,2.88)	130	0.244	0.606	0.189	(0.008, 0.492)
MIP-1β	249.49 ± 108.79	278.99 ± 59.86	-0.978	0.339	0.606	-0.35	(-1.003, 0.308)
PDGF-ββ	459.69(384.6,800.96)	666.46(504.43,820.99)	138	0.358	0.606	0.149	(0.003, 0.477)
RANTES	10076.42 ± 5957.09	12375.13 ± 3734.86	-1.439	0.159	0.583	-0.477	(-1.134, 0.186)
SCF	82.44(62.85,89.85)	70.46(57.48,88.22)	146	0.5	0.708	0.108	(0.003, 0.455)
SCGF-β	73671.30 ± 28465.09	87247.93 ± 25679.55	-1.52	0.137	0.583	-0.505	(-1.162, 0.16)
SDF-1a	759.69(600.96,1478.47)	1432.23(869.13,1852.28)	90	0.017	0.15	0.391	(0.083, 0.659)
TNF-a	115.51 ± 52.85	129.83 ± 38.91	-0.95	0.348	0.606	-0.315	(-0.968, 0.341)
TNF-β	339.46 ± 206.15	382.61 ± 114.65	-0.753	0.459	0.696	-0.269	(-0.921, 0.386)
TRAIL	49.49 ± 17.14	55.85 ± 14.97	-1.203	0.237	0.606	-0.399	(-1.053, 0.261)

The results showed no significant differences (P > 0.05) in the levels of β-NGF, FGF basic, G-CSF, GRO-α, HGF, IFN-α2, IFNγ, IL-1α, IL-1β, IL-1ra, IL-2, IL-2Ra, IL-3, IL-4, IL-6, IL-7, IL-8, IL-9, IL-10, IL-12(p70), IL-12(p40), IL-13, IL-16, IL-17A, IL-18, LIF, M-CSF, MCP-3, MIF, MIG, MIP-1α, MIP-1β, PDGF-ββ, RANTES, SCF, SCGF-β, TNF-α, TNF-β, and TRAIL between the two groups. In contrast, significant differences (P < 0.05) were observed for CTACK, Eotaxin, IP-10, MCP-1, and SDF-1α. However, following false discovery rate (FDR) correction, these differences were no longer statistically significant (FDR > 0.05), indicating that the initial findings did not survive multiple testing adjustment.

### 3.3 Comparison of serum cytokine levels between healthy controls and LTBI groups following PPD inoculation

Following PPD vaccination, serum cytokine levels were compared between the normal control group and the latent infection group. Nine cytokines, including CTACK, Eotaxin, G-CSF, IFNγ, IL-3, IL-7, RANTES, SCGF-β, and TNF-β, exhibited normal distribution (P>0.05) and were analyzed using independent sample t-tests. The remaining 35 cytokines, which did not conform to normal distribution (P<0.05), were subjected to Wilcoxon rank-sum test analysis, as detailed in **Table 3**.

**Table 3:** Comparison of cytokine levels between the control and LTBI groups following PPD administration.

Cytokine	Normal group mean ± SD/Median [P25-P75]	LTBI group mean ± SD/Median [P25-P75]	Test statistic (t/U)	p value	q value	Effect size (Cohen’s d/r)	95% CI for effect size
β-NGF	0.54(0,2.31)	2.4(1.84,3.22)	77	0.005	0.022	0.46	(0.182, 0.71)
CTACK	715.56 ± 295.56	727.3 ± 421.56	-0.095	0.925	0.925	-0.031	(-0.682, 0.619)
Eotaxin	52.56 ± 23.07	99.61 ± 59.88	-2.971	0.005	0.022	-0.986	(-1.67, -0.29)
FGF basic	19.32(0.38,34.55)	32.65(30.44,37.47)	104.5	0.05	0.122	0.319	(0.038, 0.618)
G-CSF	51.83 ± 23.91	79.4 ± 20.36	-3.785	0.001	0.011	-1.256	(-1.962, -0.535)
GRO-a	958.9(590.89,1160.84)	1205.17(997.65,1259.28)	87.5	0.014	0.039	0.403	(0.124, 0.659)
HGF	414.54(307.42,506.91)	358.68(305.51,441.3)	137.5	0.35	0.453	0.151	(0.005, 0.462)
IFN-a2	3.7(0,6.17)	5.57(4.95,6.37)	117.5	0.116	0.211	0.256	(0.013, 0.579)
IFN-γ	38.16 ± 9.7	40.14 ± 5.93	-0.766	0.449	0.534	-0.254	(-0.906, 0.401)
IL-10	2.66(0.64,12.6)	12.76(11.45,15.39)	82	0.008	0.027	0.433	(0.135, 0.693)
IL-12(p40)	39.79(31.64,51.59)	53.16(40.51,59.4)	108	0.063	0.132	0.304	(0.028, 0.584)
IL-12(p70)	4.07(0.28,7.55)	7.17(5.93,8.05)	124	0.176	0.235	0.22	(0.013, 0.54)
IL-13	2.26(1.35,3.87)	3.32(1.86,5.09)	115.5	0.107	0.211	0.262	(0.023, 0.556)
IL-16	38.67(32.71,49.71)	36.7(33.66,45.36)	160	0.806	0.825	0.038	(0.003, 0.401)
IL-17A	6.38(3.77,18.28)	19.27(17.29,21.72)	76.5	0.005	0.022	0.46	(0.165, 0.694)
IL-18	40.41(31.46,51.36)	52.49(37.25,58.5)	120.5	0.145	0.211	0.237	(0.018, 0.517)
IL-1a	17.46(9.19,38.25)	40.62(35.86,42.98)	75	0.004	0.022	0.47	(0.191, 0.707)
IL-1β	2.98(1.81,5.86)	6.47(5.39,7.01)	71.5	0.003	0.022	0.484	(0.184, 0.725)
IL-1ra	202.78(131.46,270.88)	183.09(153.68,231.81)	158.5	0.77	0.825	0.046	(0.005, 0.41)
IL-2	0(0,5.98)	6.65(5.98,6.65)	65	0.001	0.011	0.538	(0.256, 0.779)
IL-2Ra	50.57(42.61,71.94)	54.96(43.77,66.26)	160	0.806	0.825	0.038	(0.003, 0.376)
IL-3	0.49 ± 0.4	0.84 ± 0.41	-2.603	0.013	0.038	-0.864	(-1.539, -0.177)
IL-4	0.64(0.1,1.57)	1.8(1.67,1.95)	48.5	0	0	0.603	(0.37, 0.786)
IL-6	0(0,2.2)	2.26(1.79,2.97)	82	0.007	0.026	0.441	(0.158, 0.68)
IL-7	19.68 ± 11.32	26.42 ± 13.89	-1.582	0.123	0.211	-0.525	(-1.183, 0.14)
IL-8	8.38(6.26,10.92)	9.99(7.82,11.72)	122	0.158	0.217	0.229	(0.018, 0.536)
IL-9	293.15(132.36,445.38)	403.67(300.59,457.2)	107	0.061	0.132	0.305	(0.038, 0.614)
IP-10	250.87(198.19,515.7)	476.19(342.99,743.79)	87	0.013	0.038	0.406	(0.093, 0.659)
LIF	22.25(13.79,64.63)	66.83(54.71,71.2)	80.5	0.007	0.026	0.441	(0.132, 0.695)
MCP-1	34.19(29.27,47.15)	17.35(13.64,22.67)	60	0.001	0.011	0.542	(0.27, 0.77)
MCP-3	1.18(0,5.09)	5.31(3.85,6.3)	93.5	0.021	0.054	0.377	(0.063, 0.671)
M-CSF	14.37(10.76,15.58)	14.74(12.56,16.69)	139	0.374	0.47	0.144	(0.008, 0.464)
MIF	364.49(263.63,529.7)	290.67(226.77,625.89)	143	0.443	0.534	0.123	(0.003, 0.442)
MIG	211.7(169.84,253.5)	244.77(211.45,409.44)	107	0.061	0.132	0.305	(0.03, 0.579)
MIP-1a	2.29(1.35,2.79)	1.5(0.91,2.8)	121	0.149	0.211	0.235	(0.008, 0.551)
MIP-1β	230.58(155.19,275.85)	226.28(208.77,254.75)	160	0.806	0.825	0.038	(0.003, 0.371)
PDGF-ββ	467.56(273,676.28)	440.09(280.83,773.39)	159.5	0.794	0.825	0.04	(0.003, 0.384)
RANTES	9406.63 ± 4166.62	10237.55 ± 3591.28	-0.651	0.52	0.602	-0.216	(-0.867, 0.438)
SCF	75.57(60.55,90.15)	80.37(71.1,98.02)	119.5	0.137	0.211	0.242	(0.013, 0.53)
SCGF-β	72726.2 ± 29719.04	57689.27 ± 30668.45	1.497	0.143	0.211	0.497	(-0.167, 1.154)
SDF-1a	828.48(722.09,1330.01)	1419.24(1266.38,2132.66)	65	0.002	0.018	0.517	(0.235, 0.739)
TNF-a	120.48(70.27,137.1)	128.33(112.6,147.83)	120	0.141	0.211	0.24	(0.013, 0.519)
TNF-β	299.09 ± 120.65	353.07 ± 81.38	-1.625	0.113	0.211	-0.539	(-1.198, 0.127)
TRAIL	51.32(40.57,61.17)	42.53(33.75,55.88)	118.5	0.129	0.211	0.247	(0.013, 0.523)

**Figure 1:**
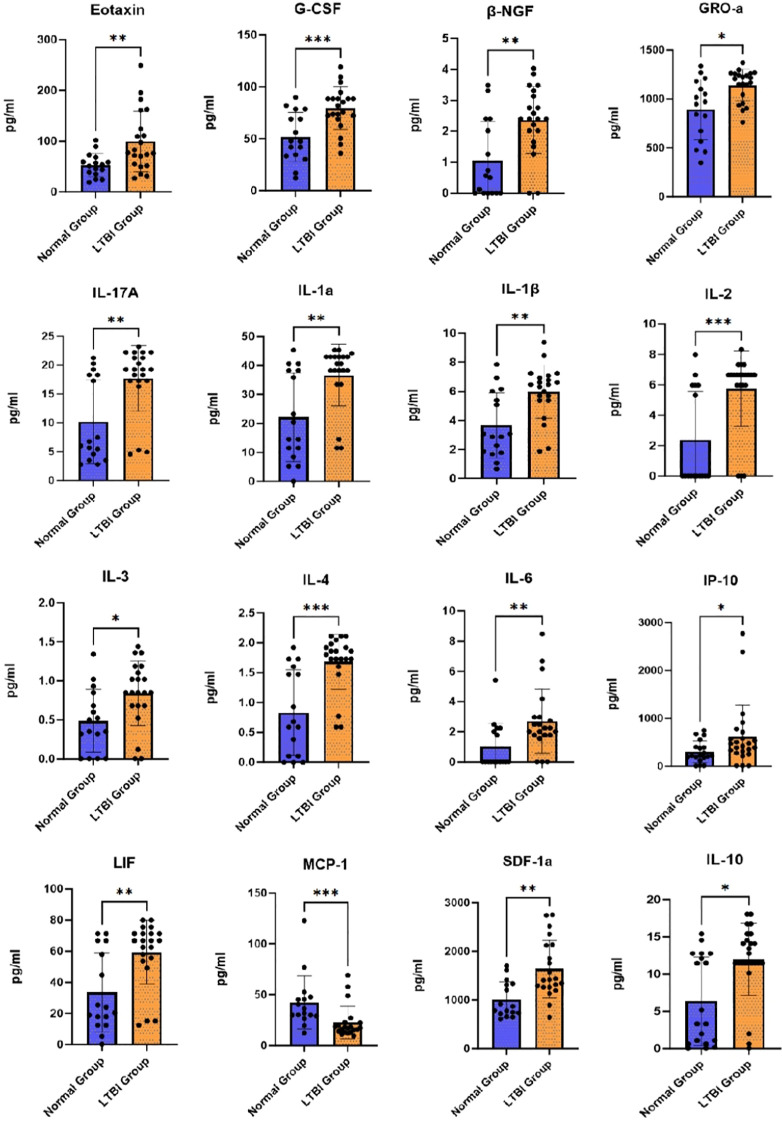
Differential cytokine levels between the control and LTBI groups following PPD administration. *p ≤ 0.05; **p ≤ 0.01; ***p ≤ 0.001.

The results revealed no significant differences in 26 cytokines: CTACK, HGF, IFN-a2, IFNγ, IL-12(p40), IL-12(p70), IL-13, IL-16, IL-18, IL-1ra, IL-2Ra, IL-7, IL-8, IL-9, M-CSF, MIF, MIG, MIP-1a, MIP-1β, PDGF-ββ, RANTES, SCF, SCGF-β, TNF-a, TNF-β, and TRAIL (P>0.05), as shown in **Table 3**. Significant differences were observed in 18 cytokines: β-NGF, Eotaxin, G-CSF, GRO-a, IL-10, IL-17A, IL-1a, IL-1β, IL-2, IL-3, IL-4, IL-6, IP-10, LIF, MCP-1, FGF basic, MCP-3, and SDF-1a (P<0.05), as detailed in **Table 3**. After FDR correction, FGF basic and MCP-3 were excluded (FDR>0.05), while significant differences were retained for β-NGF, Eotaxin, G-CSF, GRO-a, IL-10, IL-17A, IL-1a, IL-1β, IL-2, IL-3, IL-4, IL-6, IP-10, LIF, MCP-1, and SDF-1a (P<0.05, FDR<0.05), as illustrated in **Figure 1**.

### 3.4 Comparison of serum cytokine levels across groups before and after PPD administration

Changes in serum cytokine levels were compared across the healthy control group, latent tuberculosis infection (LTBI) group, and total study population before and after PPD skin test administration. For variables with a normal distribution (P > 0.05), paired-samples t-tests were performed. For non-normally distributed data (P < 0.05), the Wilcoxon signed-rank test was used for analysis, as detailed in **Table 4**.

**Table 4:** Comparison of cytokine levels before and after PPD administration.

Cytokine	Sample size (n)	PPD before (Mean ± SD/Median [P25-P75])	PPD after (Mean ± SD/Median [P25-P75])	Test statistic (Z/t)	p value	q value	Effect size (Cohen’s d/r)	95% CI for effect size
b-NGF	16	1.15 ± 1.36	1.05 ± 1.26	0.811	0.43	0.654	0.203	(-0.296,0.695)
	21	1.78 ± 1.62	2.36 ± 1.08	-1.561	0.134	0.433	-0.341	(-0.777, 0.104)
	37	1.19(0-2.76)	2.02(0.31-2.76)	-.814b	0.416	0.654	0.139	(0.005, 0.438)
CTACK	16	684.22 ± 268.14	715.56 ± 295.56	-0.453	0.657	0.85	-0.113	(-0.603,0.38)
	21	1019.64 ± 477.4	727.3 ± 421.56	2.567	0.018	0.149	0.56	(0.093, 1.015)
	37	874.59 ± 430.06	722.22 ± 367.64	2.024	0.05	0.275	0.333	(-0.001, 0.662)
Eotaxin	16	62.92 ± 57.57	52.56 ± 23.07	0.991	0.338	0.611	0.248	(-0.254,0.742)
	21	129.37 ± 91.33	99.61 ± 59.88	1.402	0.176	0.494	0.306	(-0.136, 0.74)
	37	77.96(37.59-124.29)	68.22(44.6-99.54)	-1.094b	0.274	0.554	0.179	(0.01, 0.462)
FGF basic	16	23.61 ± 24.14	22.37 ± 24.57	0.791	0.441	0.654	0.198	(-0.3,0.69)
	21	32.65(1.53-36.42)	32.65(30.44-37.47)	-1.634b	0.102	0.364	0.353	(0.023, 0.695)
	37	32.65(1.53-36.42)	32.65(9.26-34.82)	-.912b	0.362	0.627	0.148	(0.006, 0.437)
G-CSF	16	52.11 ± 24.77	51.83 ± 23.91	0.121	0.905	0.941	0.03	(-0.46,0.52)
	21	50.81 ± 20.1	79.4 ± 20.36	-4.391	0	0	-0.958	(-1.47, -0.43)
	37	51.37 ± 21.92	67.48 ± 25.7	-3.6	0.001	0.022	-0.592	(-0.938, -0.238)
GRO-a	16	827.36(132.46-1219.17)	958.9(590.89-1160.84)	-1.224b	0.221	0.523	0.298	(0.01, 0.665)
	21	1067.22 ± 210.92	1139.7 ± 162.2	-1.332	0.198	0.512	-0.291	(-0.724, 0.15)
	37	971.78(808.15-1246.15)	1145.34(893.16-1238.35)	-1.474b	0.14	0.433	0.241	(0.017, 0.515)
HGF	16	426.42 ± 133.51	413.18 ± 105.38	0.418	0.682	0.866	0.104	(-0.389,0.594)
	21	451.69 ± 100.24	384.49 ± 100.95	2.601	0.017	0.149	0.568	(0.1, 1.024)
	37	440.76 ± 114.77	396.9 ± 102.45	2.163	0.037	0.244	0.356	(0.021, 0.686)
IFN-a2	16	0(0-6.17)	3.7(0-6.17)	-.770b	0.441	0.654	0.178	(0, 0.588)
	21	4.74(0-6.37)	5.57(4.95-6.37)	-1.493b	0.136	0.433	0.321	(0.019, 0.642)
	37	4.74(0-6.37)	5.57(0.76-6.37)	-1.716b	0.086	0.355	0.28	(0.027, 0.526)
IFN-γ	16	36.96 ± 15.58	38.16 ± 9.7	-0.38	0.709	0.89	-0.095	(-0.585,0.398)
	21	41.71 ± 9.9	40.14 ± 5.93	0.62	0.543	0.763	0.135	(-0.296, 0.563)
	37	39.65 ± 12.7	39.28 ± 7.73	0.189	0.851	0.941	0.031	(-0.291, 0.353)
IL-10	16	2.21(0.64-12.76)	2.66(0.64-12.6)	-.903b	0.366	0.627	0.216	(0.006, 0.619)
	21	11.45(0.76-13.42)	12.76(11.45-15.39)	-1.478b	0.139	0.433	0.319	(0.015, 0.661)
	37	3.1(0.64-12.76)	11.45(1.54-14.07)	-.689b	0.491	0.697	0.11	(0.004, 0.408)
IL-12(p40)	16	46.1 ± 21.21	42.44 ± 15.13	1.102	0.288	0.559	0.276	(-0.229,0.771)
	21	43.21 ± 17.87	51.23 ± 15.72	-2.144	0.044	0.253	-0.468	(-0.914, -0.011)
	37	44.46 ± 19.16	47.43 ± 15.88	-1.096	0.28	0.554	-0.18	(-0.504, 0.146)
IL-12(p70)	16	3.85(0.04-7.42)	4.07(0.28-7.55)	-.734b	0.463	0.664	0.175	(0.007, 0.596)
	21	4.3 ± 3.66	6.33 ± 2.88	-2.668	0.015	0.149	-0.582	(-1.04, -0.112)
	37	5.68(0.15-6.68)	6.68(1.8-7.67)	-2.460b	0.014	0.149	0.403	(0.083, 0.617)
IL-13	16	2.02 ± 1.91	2.52 ± 1.67	-0.901	0.382	0.631	-0.225	(-0.718,0.275)
	21	3.48 ± 3.29	3.86 ± 2.76	-0.487	0.631	0.85	-0.106	(-0.534, 0.324)
	37	2.03(1.54-3.06)	2.88(1.61-4.45)	-1.180b	0.238	0.524	0.194	(0.009, 0.509)
IL-16	16	42.89(35.4-56.62)	38.67(32.71-49.71)	-1.647b	0.1	0.364	0.405	(0.019, 0.758)
	21	42.24 ± 15.13	39.84 ± 7.58	0.769	0.451	0.661	0.168	(-0.265, 0.597)
	37	41.21(32.72-56.63)	37.75(33.66-46.7)	-1.572b	0.116	0.403	0.257	(0.023, 0.536)
IL-17A	16	10.09 ± 7.91	10.19 ± 7.29	-0.228	0.823	0.937	-0.057	(-0.546,0.434)
	21	18.28(4.93-20.25)	19.27(17.29-21.72)	-1.326b	0.185	0.494	0.285	(0.015, 0.653)
	37	6.56(4.75-19.76)	18.28(5.56-20.25)	-1.129b	0.259	0.551	0.186	(0.008, 0.454)
IL-18	16	49.24 ± 24.99	44.06 ± 17.44	1.382	0.187	0.494	0.346	(-0.165,0.845)
	21	64.1 ± 21.09	53.31 ± 22.01	2.149	0.044	0.253	0.469	(0.012, 0.915)
	37	57.67 ± 23.73	49.31 ± 20.43	2.557	0.015	0.149	0.42	(0.081, 0.754)
IL-1a	16	22.45 ± 17.07	22.09 ± 15.34	0.205	0.84	0.94	0.051	(-0.44,0.541)
	21	35.86(11.51-42.98)	40.62(35.86-42.98)	-1.187b	0.235	0.524	0.255	(0.008, 0.611)
	37	33.12(9.96-40.62)	38.25(14.52-42.98)	-.778b	0.436	0.654	0.126	(0.005, 0.401)
IL-1b	16	3.57 ± 2.96	3.67 ± 2.24	-0.29	0.775	0.919	-0.073	(-0.562,0.419)
	21	5.06 ± 2.78	5.98 ± 1.82	-1.825	0.083	0.355	-0.398	(-0.839, 0.051)
	37	4.42 ± 2.92	4.98 ± 2.3	-1.744	0.09	0.36	-0.287	(-0.614, 0.044)
IL-1ra	16	223.75 ± 98.52	217.87 ± 102.05	0.308	0.763	0.919	0.077	(-0.415,0.566)
	21	199.35 ± 74.78	198.19 ± 55.19	0.082	0.935	0.957	0.018	(-0.41, 0.445)
	37	209.9 ± 85.44	206.7 ± 78.28	0.282	0.78	0.919	0.046	(-0.276, 0.368)
IL-2	16	0(0-7.32)	0(0-5.98)	-2.375b	0.018	0.149	0.572	(0.34, 0.727)
	21	5.98(0-7.32)	6.65(5.98-6.65)	-1.220b	0.222	0.523	0.261	(0.006, 0.592)
	37	0.05(0-7.32)	5.98(0-6.65)	.000b	1	1	0	(0.003, 0.371)
IL-2Ra	16	55.02 ± 20.38	56.1 ± 18.91	-0.258	0.8	0.926	-0.064	(-0.554,0.427)
	21	57.77 ± 21.01	57.97 ± 20.24	-0.035	0.972	0.979	-0.008	(-0.435, 0.42)
	37	56.58 ± 20.5	57.16 ± 19.43	-0.159	0.875	0.941	-0.026	(-0.348, 0.296)
IL-3	16	0.42 ± 0.38	0.49 ± 0.4	-1.004	0.331	0.607	-0.251	(-0.745,0.251)
	21	0.42 ± 0.48	0.84 ± 0.41	-3.628	0.002	0.038	-0.792	(-1.276, -0.292)
	37	0.42 ± 0.43	0.69 ± 0.44	-3.487	0.001	0.022	-0.573	(-0.918, -0.222)
IL-4	16	0.94 ± 0.94	0.83 ± 0.72	1.119	0.281	0.554	0.28	(-0.225,0.775)
	21	1.34 ± 0.75	1.68 ± 0.46	-2.236	0.037	0.244	-0.488	(-0.936, -0.029)
	37	1.16 ± 0.85	1.31 ± 0.72	-1.455	0.154	0.462	-0.239	(-0.564, 0.089)
IL-6	16	0(0-2.91)	0(0-2.2)	-.943b	0.345	0.615	0.21	(0, 0.617)
	21	1.92 ± 2	2.7 ± 2.14	-2.212	0.039	0.245	-0.483	(-0.93, -0.025)
	37	1.56(0-2.97)	2.02(0-2.49)	-1.186b	0.236	0.524	0.193	(0.008, 0.454)
IL-7	16	17.02 ± 13.34	19.68 ± 11.32	-0.945	0.36	0.627	-0.236	(-0.729,0.265)
	21	22.82 ± 16.56	26.42 ± 13.89	-0.892	0.383	0.631	-0.195	(-0.624, 0.24)
	37	20.31 ± 15.33	23.51 ± 13.12	-1.245	0.221	0.523	-0.205	(-0.529, 0.122)
IL-8	16	8.87(5.61-11.72)	8.38(6.26-10.92)	-.155b	0.877	0.941	0.032	(0, 0.589)
	21	9.59 ± 4.08	11.05 ± 5.01	-1.368	0.186	0.494	-0.299	(-0.733, 0.142)
	37	8.85(6.78-10.77)	8.87(7.06-11.33)	-.422b	0.673	0.862	0.069	(0.005, 0.397)
IL-9	16	328.57 ± 236.26	292.88 ± 147.68	1.045	0.312	0.588	0.261	(-0.241,0.756)
	21	389.05 ± 152.99	391.5 ± 104.7	-0.064	0.95	0.965	-0.014	(-0.441, 0.414)
	37	362.9 ± 192.83	348.85 ± 132.78	0.536	0.595	0.818	0.088	(-0.235, 0.41)
IP-10	16	338.37 ± 204.38	350.83 ± 201.66	-0.301	0.767	0.919	-0.075	(-0.565,0.417)
	21	507.01(327.19-830.82)	476.19(342.99-743.79)	-.122b	0.903	0.941	0.023	(0, 0.456)
	37	372.94(268.78-661.47)	389.55(236.75-615.36)	-.098b	0.922	0.951	0.015	(0.002, 0.375)
LIF	16	35.58 ± 32.03	33.35 ± 25.32	0.8	0.436	0.654	0.2	(-0.298,0.692)
	21	47.18 ± 33.19	59.39 ± 20.53	-1.907	0.071	0.323	-0.416	(-0.858, 0.035)
	37	42.16 ± 32.76	48.13 ± 25.93	-1.504	0.141	0.433	-0.247	(-0.573, 0.082)
MCP-1(MCAF)	16	51.48 ± 27.26	42.35 ± 26.22	1.74	0.102	0.364	0.435	(-0.085,0.942)
	21	24.21(18.14-39.41)	17.35(13.64-22.67)	-2.624b	0.009	0.119	0.569	(0.205, 0.865)
	37	40.28 ± 25.22	31.22 ± 22.89	2.808	0.008	0.117	0.462	(0.119, 0.798)
MCP-3	16	0.96(0-3.2)	1.18(0-5.09)	-1.245b	0.213	0.523	0.3	(0, 0.657)
	21	2.01 ± 1.89	4.74 ± 2.28	-5.123	0	0	-1.118	(-1.658, -0.56)
	37	1.8(0-3.38)	4.41(0-6.19)	-3.925b	0	0	0.643	(0.441, 0.758)
M-CSF	16	14.39 ± 5.05	14 ± 4.24	0.32	0.753	0.919	0.08	(-0.412,0.57)
	21	14.51 ± 5.19	15.45 ± 4.21	-0.87	0.395	0.636	-0.19	(-0.619, 0.244)
	37	14.46 ± 5.05	14.83 ± 4.23	-0.456	0.651	0.85	-0.075	(-0.397, 0.248)
MIF	16	340.03(263.91-522.52)	364.49(263.63-529.7)	-.155b	0.877	0.941	0.032	(0.006, 0.552)
	21	482.35 ± 285.92	444.98 ± 315.23	0.479	0.637	0.85	0.104	(-0.326, 0.532)
	37	401.11(270.44-573.11)	333.04(231.75-559.07)	-.460b	0.645	0.85	0.074	(0.007, 0.402)
MIG	16	238.06(187.57-314.65)	211.7(169.84-253.5)	-1.931b	0.053	0.279	0.476	(0.063, 0.757)
	21	286.13(172.26-453.59)	244.77(211.45-409.44)	-.226b	0.821	0.937	0.046	(0, 0.493)
	37	245.56(179.73-371.71)	223.55(177.89-289.57)	-1.147b	0.251	0.543	0.187	(0.012, 0.496)
MIP-1a	16	2.16(1.71-2.81)	2.29(1.35-2.79)	-.738b	0.46	0.664	0.177	(0, 0.602)
	21	1.79(1.39-2.88)	1.5(0.91-2.8)	-.365b	0.715	0.89	0.076	(0.008, 0.54)
	37	1.92(1.55-2.83)	1.69(1.16-2.74)	-.786b	0.432	0.654	0.127	(0.005, 0.427)
MIP-1b	16	249.49 ± 108.79	225.17 ± 68.88	1.139	0.273	0.554	0.285	(-0.22,0.781)
	21	278.99 ± 59.86	236.67 ± 55.32	2.033	0.055	0.279	0.444	(-0.01, 0.888)
	37	266.23 ± 84.51	231.69 ± 60.92	2.324	0.026	0.202	0.382	(0.046, 0.714)
PDGF-bb	16	727.64 ± 858.82	604.04 ± 622.01	1.134	0.275	0.554	0.283	(-0.221,0.779)
	21	670.28 ± 355.94	508.78 ± 305.18	1.927	0.068	0.321	0.421	(-0.031, 0.863)
	37	695.08 ± 615.26	549.98 ± 463.94	2.196	0.035	0.244	0.361	(0.026, 0.692)
RANTES	16	10076.42 ± 5957.09	9406.63 ± 4166.62	0.573	0.575	0.799	0.143	(-0.352,0.633)
	21	12375.13 ± 3734.86	10237.55 ± 3591.28	1.765	0.093	0.361	0.385	(-0.063, 0.825)
	37	11381.09 ± 4885.56	9878.24 ± 3817.45	1.767	0.086	0.355	0.29	(-0.041, 0.618)
SCF	16	82.44(62.85-89.85)	75.57(60.55-90.15)	-.259b	0.796	0.926	0.058	(0.006, 0.55)
	21	76.31 ± 30.24	85.73 ± 19.11	-1.422	0.171	0.491	-0.31	(-0.745, 0.132)
	37	75.99 ± 26.62	81.52 ± 18.13	-1.243	0.222	0.523	-0.204	(-0.529, 0.123)
SCGF-b	16	73671.3 ± 28465.09	72726.2 ± 29719.04	0.131	0.898	0.941	0.033	(-0.458,0.522)
	21	87247.93 ± 25679.55	57689.27 ± 30668.45	4.36	0	0	0.952	(0.425, 1.462)
	37	81376.95 ± 27394.53	64191.72 ± 30782.71	3.166	0.003	0.05	0.521	(0.174, 0.861)
SDF-1a	16	759.69(600.96-1478.47)	828.48(722.09-1330.01)	-.465b	0.642	0.85	0.11	(0.006, 0.615)
	21	1593.07 ± 965.85	1640.42 ± 592.19	-0.216	0.831	0.938	-0.047	(-0.474, 0.381)
	37	1159.96(717.73-1698.95)	1310.31(828.48-1659.56)	-.173b	0.862	0.941	0.027	(0.005, 0.38)
TNF-a	16	127.35(61.28-160.92)	120.48(70.27-137.1)	-.982b	0.326	0.606	0.239	(0.006, 0.68)
	21	129.83 ± 38.91	131.08 ± 24.02	-0.137	0.892	0.941	-0.03	(-0.457, 0.398)
	37	123.64 ± 45.35	121.56 ± 30.92	0.317	0.753	0.919	0.052	(-0.271, 0.374)
TNF-b	16	339.46 ± 206.15	299.09 ± 120.65	1.074	0.3	0.574	0.268	(-0.235,0.763)
	21	382.61 ± 114.65	353.07 ± 81.38	0.905	0.376	0.631	0.198	(-0.237, 0.627)
	37	363.95 ± 159.62	329.73 ± 102.37	1.407	0.168	0.491	0.231	(-0.097, 0.556)
TRAIL	16	49.49 ± 17.14	54.75 ± 18.02	-1.249	0.231	0.524	-0.312	(-0.81,0.195)
	21	55.85 ± 14.97	46.5 ± 15.76	1.939	0.067	0.321	0.423	(-0.029, 0.865)
	37	53.1 ± 16.03	50.07 ± 17.04	0.876	0.387	0.631	0.144	(-0.181, 0.467)

**Figure 2:**
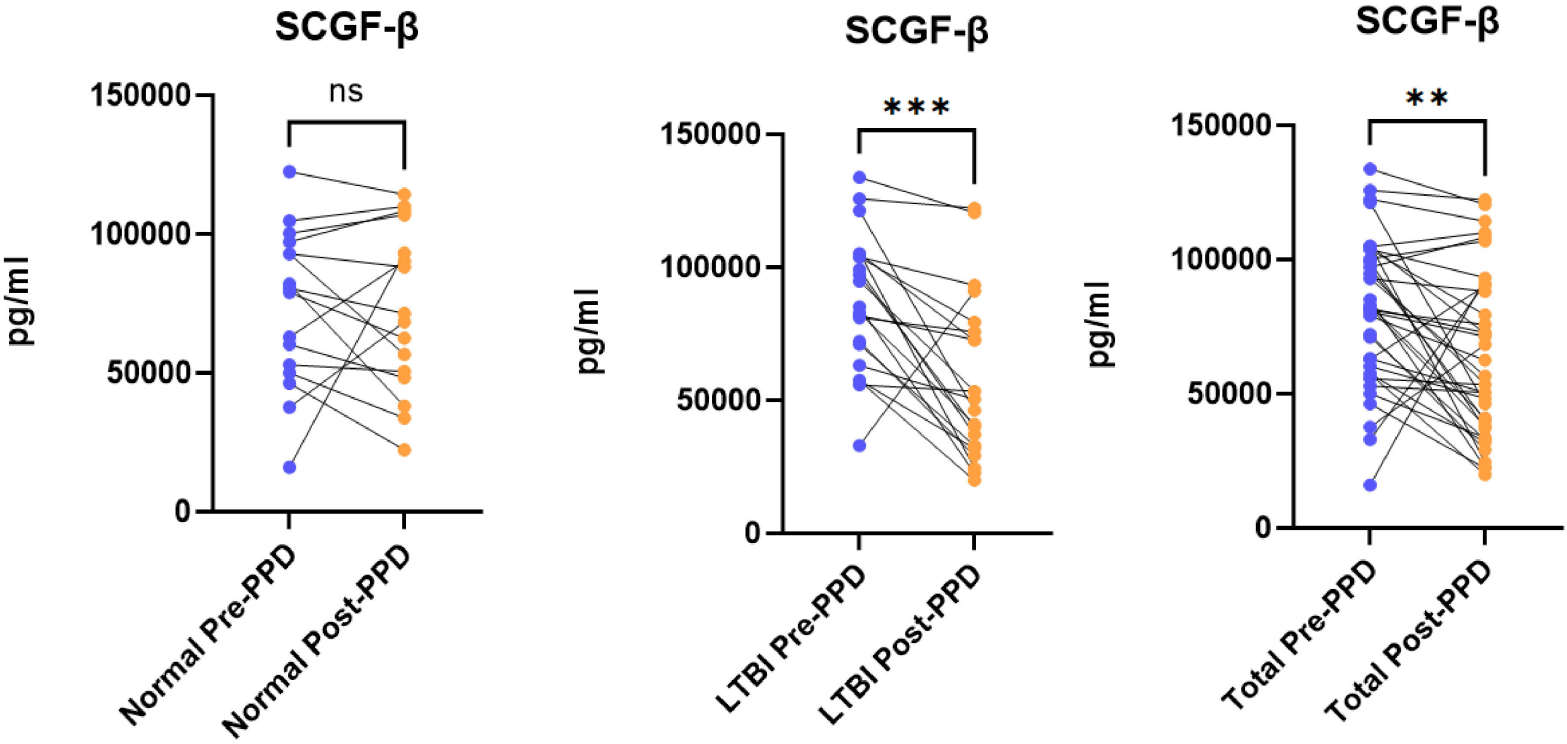
Comparison of SCGF-β levels before and after PPD administration. ns p > 0.05; **p ≤ 0.01; ***p ≤ 0.001.

**Figure 3:**
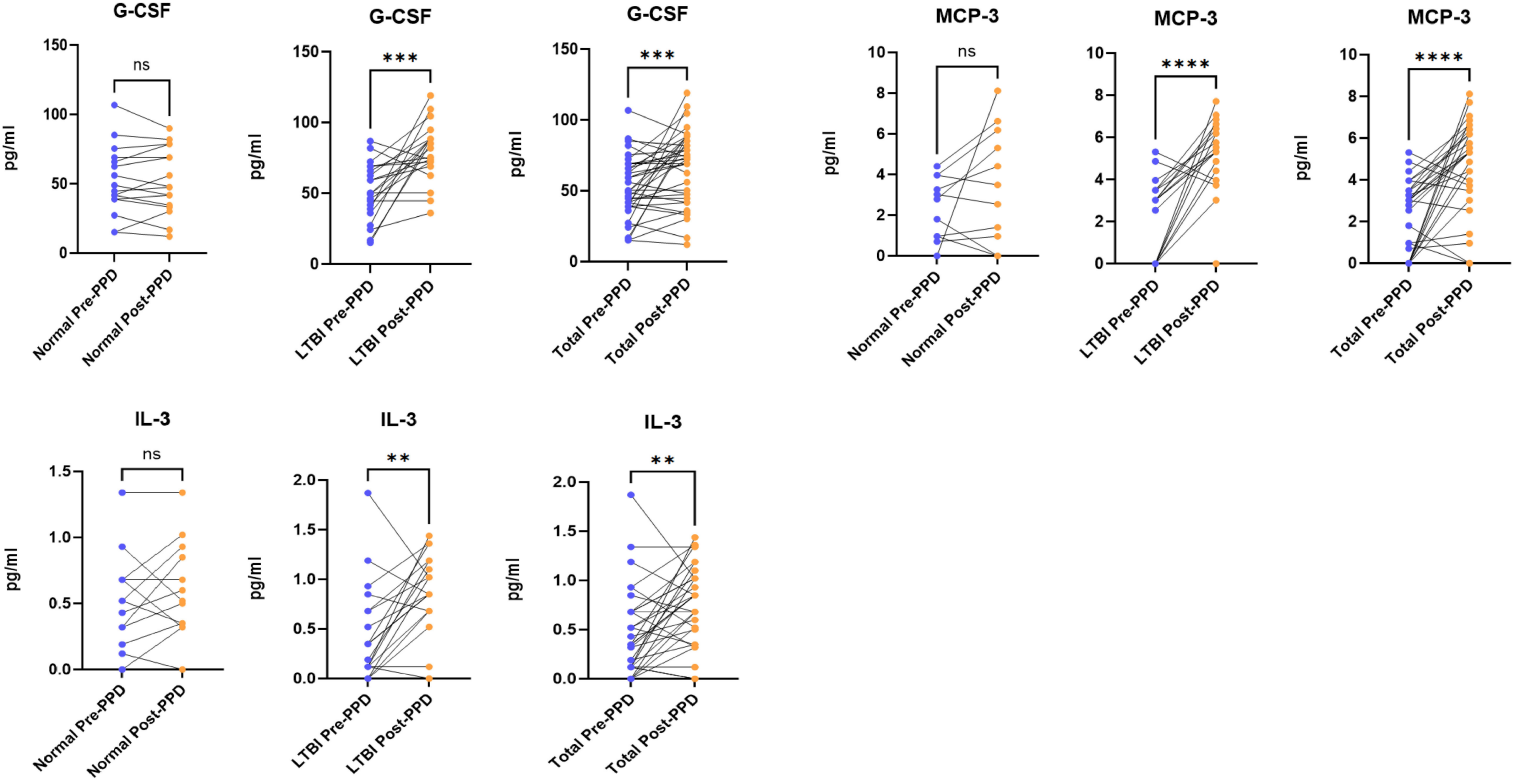
Comparison of G-CSF, MCP-3 and IL-3 levels before and after PPD administration. ns p > 0.05; **p ≤ 0.01; ***p ≤ 0.001.

Comparative analysis of serum cytokine profiles before and after PPD skin test administration revealed significant alterations in individuals with latent tuberculosis infection (LTBI). Among the 44 cytokines analyzed, twelve showed statistically significant differences (P < 0.05): CTACK, G-CSF, HGF, IL-18, MCP-1, SCGF-β, IL-12(p40), IL-12(p70), IL-6, MCP-3, IL-3, and IL-4. Specifically, five cytokines—CTACK, SCGF-β, HGF, MCP-1, and IL-18—exhibited decreased levels, while seven cytokines—G-CSF, IL-12(p40), IL-12(p70), MCP-3, IL-6, IL-3, and IL-4—showed increased levels. Following false discovery rate (FDR) correction, eight cytokines (CTACK, HGF, IL-18, MCP-1, IL-12(p40), IL-12(p70), IL-6, and IL-4) no longer remained statistically significant (FDR > 0.05), leaving only SCGF-β, G-CSF, MCP-3, and IL-3 as significantly differentially expressed (P < 0.05, FDR < 0.05). Detailed results are presented in **Figures 2** and **3**.

#### 3.4.1 Downregulation of SCGF-β in the serum of individuals with latent tuberculosis infection before and after PPD skin test administration

In the normal control group, serum levels of SCGF-β exhibited minimal variation before and after PPD skin test administration. Among individuals with latent tuberculosis infection (LTBI), a significant reduction in serum SCGF-β levels was observed following PPD testing (P < 0.05, FDR < 0.05). A similar statistically significant decrease was also evident in the total study population (P < 0.05, FDR < 0.05). Detailed results are presented in **Figure 2**.

#### 3.4.2 Upregulation of G-CSF, MCP-3, and IL-3 in the serum of individuals with latent tuberculosis infection before and after PPD skin test administration

The levels of three cytokines—G-CSF, MCP-3, and IL-3—exhibited minimal variation in the general population before and after PPD skin test administration. In contrast, among individuals with latent tuberculosis infection (LTBI), serum concentrations of all three cytokines showed a significant increase, with statistically significant differences (P < 0.05, FDR < 0.05). A similar elevation was also observed in the total study population (P < 0.05, FDR < 0.05). Detailed results are presented in **Figure 3**.

## 4 Discussion

Latent tuberculosis infection (LTBI) represents a state in which *Mycobacterium tuberculosis* persists in the host without causing active disease, maintained by a delicate balance between immune activation and regulation. This equilibrium is orchestrated through precise immunomodulatory mechanisms that prevent tissue damage while controlling bacterial persistence. Central to this regulation is the differentiation of T helper (Th) cell subsets, determined by local cytokine milieus and co-stimulatory signals from antigen-presenting cells. CD4^+^ T cells differentiate along specific pathways: IL-12 drives Th1 polarization, IL-4 promotes Th2 differentiation, and RORγt facilitates Th17 lineage commitment (Zhou and Chong, 2009). CD8^+^ T cells, in contrast, develop into cytotoxic T lymphocytes (CTLs) capable of eliminating intracellular pathogens (Kaech and Cui, 2012; Iwasaki and Medzhitov, 2015; Mu et al., 2022). IL-2, predominantly secreted by activated CD4^+^ T cells, supports the proliferation and differentiation of both CD4^+^ and CD8^+^ T cells, playing a central role in adaptive immune responses (Gaffen and Liu, 2004; Mu et al., 2022). B cells also interact with Th subsets to maintain immune homeostasis, particularly in LTBI.

In this study, we observed that PPD administration induced a synergistic immune response in individuals with latent tuberculosis infection (LTBI), which specifically involved four cytokines: SCGF-β, G-CSF, MCP-3, and IL-3. Furthermore, it may modulate the dynamic expression of additional cytokines, including IL-2, IL-4, IL-6, IL-10, IL-17A, IL-1α, IL-1β, IP-10, LIF, SDF-1α, β-NGF, Eotaxin, GRO-α, and MCP-1. This immunomodulatory mechanism likely reflects a dynamic equilibrium between pro-inflammatory and anti-inflammatory signaling pathways, thereby enabling effective bacterial suppression while minimizing host tissue damage. Th1 and Th17 cells are well-established mediators of protective immunity against *M. tuberculosis (*Salgame, 2005; Torrado and Cooper, 2010; Ye et al., 2012). Classical studies link Th1 cells to granulomatous control of mycobacterial infection, with IFN-γ and TNF serving as hallmark Th1 cytokines that activate macrophage antimicrobial mechanisms (Cooper et al., 1993; Flynn et al., 1995; Mayer-Barber and Barber, 2015; Ritter et al., 2022). Interestingly, our results revealed no significant changes in serum IFN-γ, TNF-α, or TNF-β in LTBI subjects following PPD administration, whereas Th1-associated cytokines MCP-1 exhibited significant modulation.

MCP-1 (CCL2), produced by T cells and macrophages, recruits monocytes and Th cells to sites of infection and promotes granuloma integrity (Deshmane et al., 2009; Hussain et al., 2011). Elevated MCP-1 is associated with disease severity in active tuberculosis (Kasahara et al., 1994; Hussain et al., 2011), whereas in LTBI, low-level MCP-1 may maintain a Th1-biased environment (Lee et al., 2003). Our findings support this: LTBI individuals exhibited lower baseline MCP-1 than healthy controls, with a further decline after PPD stimulation, suggesting preservation of Th1 polarization and immune homeostasis.

Th17 cells, activated early in infection, secrete IL-17A to recruit neutrophils and monocytes, promoting granulopoiesis and inflammation (Korn et al., 2009; Cruz et al., 2010; Burkett et al., 2015; Su et al., 2018). In our study, PPD stimulation upregulated Th17-associated cytokines, including IL-6, IL-1β, and G-CSF. IL-6 and IL-1β drive naïve CD4^+^ T cell differentiation into Th17 and Th22 lineages, facilitating granuloma formation and host defense (Ye et al., 2012). G-CSF, induced by IL-17, regulates granulocyte production and supports neutrophil function (Lieschke and Burgess, 1992; Cormican et al., 2004). Elevated IL-6 and G-CSF responses correlate with active tuberculosis, suggesting potential utility in distinguishing latent from active disease (Zhang et al., 1994; Higgins et al., 2008; Wei et al., 2015).

Th2 responses, which mediate humoral immunity and regulate allergic inflammation, were also modulated by PPD. Cytokines such as IL-4, IL-10 and MCP-3 were significantly altered post-stimulation. IL-4 drives Th2 differentiation, while IL-10, produced by Treg cells, maintains immune tolerance and suppresses excessive Th1 activity (Chen et al., 2007; Su et al., 2018). MCP-3 (CCL7) recruits immune cells for protective immunity and has potential utility in differentiating LTBI from active tuberculosis, particularly in immunocompromised populations (Saunders et al., 2005; Ryan et al., 2007; Juno et al., 2016; Yao et al., 2017).

From a diagnostic perspective, our results underscore the complexity of interpreting tuberculin skin test (TST) and interferon-gamma release assay (IGRA) outcomes. While IGRA avoids cross-reactivity from BCG vaccination, it cannot distinguish latent from active infection. PPD stimulation, however, induced broad cytokine alterations without affecting IFN-γ within 48–72 hours, suggesting potential for developing cytokine-based diagnostic tools to complement existing assays.

Although this study provides insights into the differential regulation of immune responses in individuals with latent tuberculosis infection (LTBI) following purified protein derivative (PPD) vaccination, it remains at a preliminary and exploratory stage. The temporal relationship between the 48- to 72-hour post-vaccination period and underlying immune dynamics has not been clearly established, which introduces several limitations. First, the relatively small sample size and potential batch effects associated with multiplex cytokine assays may compromise the generalizability and reliability of the findings. Future studies should expand to multicenter cohorts with larger sample sizes and implement stringent measures to control for technical variability, thereby improving external validity. Second, the analysis was restricted to selected serum cytokines and did not include plasma cytokines or other biological matrices. Subsequent research could utilize high-throughput technologies to enable a more comprehensive profiling of immune mediators, thus advancing the understanding of how PPD vaccination modulates immune responses in LTBI. Moreover, although participants with chronic immune-related conditions such as diabetes mellitus and rheumatoid arthritis were excluded, the influence of acute inflammatory infections and other transient comorbidities was not assessed. Furthermore, while body mass index (BMI) was taken into account, additional factors—including markers of nutritional status and individual genetic variation—that may affect cytokine expression profiles were not evaluated and should be incorporated in future investigations.

In conclusion, PPD administration elicits distinct immune responses in LTBI, reflecting a finely tuned immunological environment that balances pathogen control with host tolerance. The modulation of multiple cytokines, including Th1, Th2, Th17, and regulatory factors, provides insights into LTBI immunopathology and offers potential avenues for early immune monitoring and diagnostic biomarker development.

## Data Availability

The raw data supporting the conclusions of this article will be made available by the authors, without undue reservation.
